# A Computer-Based Method for the Investigation of Human Behavior in the Iterative Chicken Game

**DOI:** 10.3389/fpsyg.2021.576404

**Published:** 2021-05-28

**Authors:** Sung-Phil Kim, Minju Kim, Jongmin Lee, Yang Seok Cho, Oh-Sang Kwon

**Affiliations:** ^1^Department of Biomedical Engineering, Ulsan National Institute of Science and Technology, Ulsan, South Korea; ^2^School of Psychology, Korea University, Seoul, South Korea

**Keywords:** artificial agent, chicken game, computational model, fairness, competitive social interaction

## Abstract

The present study develops an artificial agent that plays the iterative chicken game based on a computational model that describes human behavior in competitive social interactions in terms of fairness. The computational model we adopted in this study, named as the self-concept fairness model, decides the agent’s action according to the evaluation of fairness of both opponent and self. We implemented the artificial agent in a computer program with a set of parameters adjustable by researchers. These parameters allow researchers to determine the extent to which the agent behaves aggressively or cooperatively. To demonstrate the use of the proposed method for the investigation of human behavior, we performed an experiment in which human participants played the iterative chicken game against the artificial agent. Participants were divided into two groups, each being informed to play with either a person or the computer. The behavioral analysis results showed that the proposed method can induce changes in the behavioral pattern of human players by changing the agent’s behavioral pattern. Also, we found that participants tended to be more sensitive to fairness when they played with a human opponent than with a computer opponent. These results support that the artificial agent developed in this study will be useful to investigate human behavior in competitive social interactions.

## Introduction

When people make a decision in economic games, they are often motivated not only by material rewards (e.g., monetary self-interests) but also many other social factors when making a decision in economic games (Kahneman et al., 1986). One of the key factors that motivates one to make decisions is a sense of fairness (Rabin, 1993; Cox et al., 2008). When people feel the sense of fairness, they are often willing to sacrifice their monetary interests. As a source to invoke the sense of fairness, researchers have proposed that altruism is the pursuit of an unbiased distribution of welfare (Andreoni, 1995; Eckel and Grossman, 1996). For instance, instead of receiving a smaller payoff than an opponent in the ultimatum game, people tend to opt for an allocation so that neither player earns rewards (Slonim and Roth, 1998). Another proposed source is reciprocity that adjusts rewards or punishments depending on others’ kindness (Slonim and Roth, 1998; Cox and Deck, 2005). For instance, people tend to sacrifice their interests for the welfare of those who are kind to them (Isaac and Walker, 1988; Orbell et al., 1988), or punish those who are harmful (Roth et al., 1991; Thaler, 1999).

A number of computational models have been proposed to elucidate mechanisms of how fairness shapes human behavior during economic games (Rabin, 1993; Levine, 1998; Fehr and Schmidt, 1999; Dufwenberg and Kirchsteiger, 2004; Cox et al., 2007; Lee et al., 2015). Computational models illustrating altruism as a key factor to fairness, including the Fehr and Schmidth model (Fehr and Schmidt, 1999), aim to avoid inequality and weigh more in fair distribution of income. Yet, they do not consider including intentions behind others’ behavior. Other models, such as Rabin’s model, concentrate more on reciprocity by evaluating others’ kindnesses in terms of their intentions (Rabin, 1993). However, these approaches often encounter the difficulty in modeling complex beliefs about others’ intentions. Cox’s model relieves such complexity by focusing on emotional states evoked by others’ behaviors in lieu of complex beliefs, in order to encompass reciprocity in the model (Cox et al., 2007). Although this model offers a relatively simple way of representing reciprocity, it only considers the fairness of others. In contrast, a recent model by Lee et al. (2015) incorporates the self-concept of fairness into the model of reciprocity based on the fact that reciprocity emerges from bilateral joint action, which refers to a pair of actions taken simultaneously by both players in an economic game (Molm, 2010).

During iterative economic games, the evaluation of reciprocity by each player changes more dynamically with continuously changing behavioral patterns, while the evaluation of material payoffs changes relatively less (Komorita et al., 1991). Thus, understanding how a person continuously evaluates fairness during economic games requires systematic variation of fairness in an opponent’s behavior. However, it is challenging to generate such systematic behavior in a human opponent because explicit calculation of fairness each time a person plays the game would distort a natural sense of fairness and there would be always a possibility of human error in playing the game, making behavior inconsistent. As such, it would be more desirable to create a model-based agent that can systematically simulate behavior with various degrees of fairness. The self-concept fairness model can achieve this end by considering fairness of both players and produce human-like behavior based on interactive behavioral outcomes and rewards (Lee et al., 2015).

As a competitive economic game, we adopted an iterative chicken game to collect behavioral outcomes of both players reflecting reciprocity (Jankowski, 1990). We used the game environment developed in the previous studies (Asher et al., 2012; Lee et al., 2015), where two players each control a car rushing toward each other, then having to make a decision to avoid or rush within a time limit. A player gains a payoff when the opponent avoids first but both lose relatively more when no one avoids to the end. The chicken game is intrinsically competitive without single Nash equilibrium; a player needs to rush to increase a chance to earn more benefit, but at the same time it also increases the risk of crash. The chicken game allows us to observe dynamic behavioral patterns, rendering itself adequate to investigate human behavior in response to varied behavioral patterns of an opponent. In the chicken game, fairness plays a key role in decision-making because players do not have to rely on beliefs of others’ intention—others’ action such as rush and avoid has clear intention.

The present study aims to develop an artificial agent that can simulate decision-making with a sense of fairness in the iterative chicken game. The artificial agent is designed to make a decision based on the previously established self-concept fairness model that yields the probabilities of actions continuously updated from the past behavioral outcomes of both an opponent and the self as well as rewards each earned. The artificial agent can play the chicken game with various degrees of fairness by changing its model parameters. In order to verify the feasibility of investigations on human behavior using the developed artificial agent, we perform an experiment where human players compete with the artificial agent in the iterative chicken game. Specifically, we aim to examine how the behavior of human players changes according to changes in the agent’s behavior in terms of fairness. We also aim to find differences in sensitivity to fairness when players perceive an opponent as a human or non-human (i.e., computer-based) player.

While a number of studies have developed artificial agents that can learn to play the chicken game in a data-driven manner (van den Dries and Wiering, 2012; Silver et al., 2018), an artificial agent developed in this study is different from previous ones since it can play the chicken game with varying degrees of fairness based on the analytical model built on human behavior (i.e., the self-concept fairness model). One can tune the model parameters freely to implement certain degree of fairness in the agent. Therefore, this new artificial agent enables us to investigate how humans make a decision in competitive socioeconomic games with specific properties of the opponent’s fairness or even with dynamic changes of such fairness properties during game.

Also, many studies have investigated the effects of the awareness of an opponent as a human or computer on socioeconomic games such as prisoner’s dilemma game (Hegel et al., 2008; Krach et al., 2008). Yet, little is known about how a player’s behavioral responses differ against human vs. computer opponents in the iterative chicken game with varying degrees of fairness. This study addresses this question by using the artificial agent newly developed in the present study.

## Materials and Methods

### Computational Model of Fairness

The self-concept fairness model was proposed by Lee et al. (2015), focusing on reciprocity as a key factor of fairness in decision-making during the iterative chicken game. In this model, players continuously update the degree of reciprocity of both the self and an opponent over iterations by examining their past actions and collected payoffs and take action based on the probability function of fairness. In the chicken game used in this study, two players choose whether to avoid or rush the other in every iteration and receive a reward according to the result of both players’ behavior. The player’s goal is to maximize their sum of rewards. The reward scale for the chicken game used in our study is depicted in Table 1. The possible outcomes are both avoiding (AA), player 1 rushing and player 2 avoiding (RA), player 1 avoiding and player 2 rushing (AR), and both rushing (RR). From player 1’s point of view, the payoffs are RA > *AA* > *AR* > RR, and the cost of a crash (both rushing) is designed to be overwhelming compared to the loss of avoiding. Therefore, in order for the player to gain more payoffs, they need to take a risk of losing a lot.

**TABLE 1:** The reward scale of the chicken game employed in the present study.

		**Player 2**	
		**Avoid**	**Rush**
Player 1	Avoid	(0, 0)	(−300, 300)
	Rush	(300, −300)	(−1,000, −1,000)

*(*x,y*): reward earned by player 1 (*x*) and player 2 (*y*), respectively.*

Here, we briefly describe the implementation of the self-concept fairness model in the iterative chicken game but one can find more details regarding the model description and theoretical backgrounds in Lee et al. (2015). At each iteration, we first calculate the kindness of the self (*i*) and an opponent (*j*) for every possible action taken by the self (*a*_*i*_) as:

(1) $$f_{i}\,\left(a_{i}\right)=\frac{\pi_{j}^{\text{max}}\,\left(a_{i}\right)-E\,\left[\pi_{j}^{\text{max}}\right]}{E\,\left[\pi_{j}^{\text{max}}-\pi_{j}^{\text{min}}\right]}.$$

*f*_*i*_(*a*_*i*_) represents the kindness of the self (*i*) to the opponent (*j*) and $\pi_{j}^{\text{max}}\,\left(a_{i}\right)$ is the maximum reward possible given to the opponent when the self takes an action of *a*_*i*_. $E\,\left[\pi_{j}^{\text{max}}\right]$ is the expected maximum reward received by the opponent averaged over every possible action taken by the self. $E\,\left[\pi_{j}^{\text{max}}-\pi_{j}^{\text{min}}\right]$ is the expected value of a difference between the maximum possible reward and minimum possible reward given to the opponent, in order to normalize the degree of kindness for applications to different scales of rewards. The positive value of *f*_*i*_(*a*_*i*_) indicates that the self plays more generously toward the opponent and the negative value does that the self plays less generously. The kindness of the opponent to the self, *f*_*j*_(*a*_*j*_) is calculated in the same way.

After calculating the kindness values according to the actions taken at the *k*-th iteration, the degrees of fairness of the self (*i*) and the opponent (*j*) are updated, respectively, in the following manner:

(2) $$\begin{array}{c} F_{i}\,\left(k+1\right)=\gamma \,F_{i}\,\left(k\right)+\eta \,f_{i}\,\left(a_{i}\,\left(k\right)\right),\\ F_{j}\,\left(k+1\right)=\gamma \,F_{j}\,\left(k\right)+\eta \,f_{j}\,\left(a_{j}\,\left(k\right)\right)+\beta . \end{array}$$

The degree of fairness of the self at the (*k*+1)-th iteration, *F*_*i*_(*k* + 1), is updated from that at the previous *k*-th iteration, *F*_*i*_(*k*). γ represents the retention rate of fairness, reflecting the assumed volatile property of reciprocity. η > 0 is the learning rate controlling the speed of the update of the degree of fairness. β is an intrinsic benevolence parameter added to the kindness of others, which is positive when the self tends to perceive the opponent to be kinder, and negative when the self perceives the opponent to be more selfish (Cox et al., 2007).

With the updated degrees of fairness of both the self and the opponent, we calculate the reciprocity as follows:

(3) $$r_{i}\,\left(k+1\right)=2\,\left(\theta \,F_{j}\,\left(k+1\right)-\left(1-\theta \right)\,F_{i}\,\left(k+1\right)\right).$$

The reciprocity (*r*) is defined by a difference between the degrees of fairness between the self and the opponent. Yet, a relative weight on the opponent’s fairness, θ(0≤θ≤1), is also included to reflect a possible bias toward the self or others’ kindness; if θ > 0.5, the player cares more about the opponent’s kindness toward the self than the player’s kindness to the opponent. The reciprocity becomes positive if the player perceives that the opponent’s degree of kindness weighted by θ is higher than that of the self, and negative if lower. The weighted difference is multiplied by 2 in Eq. (3) in order to have reciprocity simply equal to the difference in fairness, (*F*_*j*_(*k*+1) – *F*_*i*_(*k*+1)), when there is no bias such that θ = 0.5 (see Lee et al., 2015).

The calculated reciprocity is then used to compute the utility function of each action as follows:

(4) $$\begin{array}{c} u_{i}\left(a_{i}\left(k+1\right)\right)=\frac{1}{\alpha }\{{\left(\sum_{n=1}^{k}\pi_{i}\left(n\right)+E\left[\pi_{i}^{a_{i}}\right]\right)}^{\alpha }+r_{i}\left(k+1\right)\\ {\left(\sum_{n=1}^{k}\pi_{j}\left(n\right)+E\left[\pi_{j}^{a_{i}}\right]\right)}^{\alpha }\}, \end{array}$$

where, $E\,\left[\pi_{i}^{a_{i}}\right]$ and $E\,\left[\pi_{j}^{a_{i}}\right]$ refer to the expected rewards given to the self (*i*) or the opponent (*j*), respectively, when the self takes action *a*_*i*_. The convexity parameter *α*, 0≤α≤1, reflects a player’s preference of equal distribution of payoffs between players. When *r*(*k* + 1) > 0, the player prefers equal distribution of payoffs with a small value of *α* but becomes indifferent to the distribution with a large value of *α*. When *r*(*k* + 1) < 0, the player prefers receiving all payoffs more strongly when *α* value is smaller than when the *α* value is large.

With the utility function of each action, we calculate the probability of taking an action of avoid, *p*(avoid), as follows:

(5) $$p\,\left(a\,v\,o\,i\,d\right)=\frac{1}{1+e^{-z}},z=u_{i}\,\left(a\,v\,o\,i\,d\right)-u_{i}\,\left(r\,u\,s\,h\right),$$

where the right-hand side function is known as the sigmoid function. If *u*_*i*_ (avoid) > *u*_*i*_ (rush), it is more likely that the player chooses to avoid at the (*k*+1)-th iteration. With *p*(avoid) and *p*(rush) = 1 – *p*(avoid), an actual action is produced using the Bernoulli process.

Let us assume that the self (*i*) took an action of avoidance, and the opponent (*j*) chose to rush. The kindness *f* of the self is calculated depending on the action taken by the self, and the payoff table. According to the payoff scale used in this chicken game, f of the self is 0.6 and that of the opponent becomes -0.6. Using this the value of the kindness, the degree of fairness *F* is updated. If we suppose that both *F* of the self and the opponent in this iteration is 0, the updated *F* of the self is 0.5, making relatively larger value of fairness, and *F* of the opponent is updated to −0.4311, leading to smaller value than that of the self. The reciprocity *r* is calculated using the updated F of the self and the opponent, and here, if we assume theta is 0.5, supposing no bias toward the self or the opponent, *r* becomes 0.9311, a positive value. From this reciprocity value *r*, the utility function of each action is computed. Assuming both sum of reward of the self and that of the opponent as 1,000, the utility function of avoid is calculated to be 1,781.7 and that of rush becomes 1,778.9. Finally, the probability of the action of avoid is calculated as 0.9427.

### Development of an Artificial Chicken Game Agent

We developed an artificial agent that plays the iterative chicken game. The decision process of the agent follows the self-concept fairness model described in section ‘‘Computational model of fairness.’’ We developed an algorithm that implements the decision process of the agent using the Matlab software (MATLAB ver. R2016a, Mathworks, Inc. Natick, MA, United States). Using this algorithm, we created a Matlab-based computer program to run the iterative chicken game. Matlab code for this program is made available for download^1^.

Specifically, the computer program of the iterative chicken game consists of three modules. The first module handles the parameters for the computational model of fairness. The user of the program can set the parameters through this module to determine the way the artificial agent makes a decision. For instance, with a certain parameter setting, the agent is more likely to exchange rewards mutually with an opponent by taking turns between rush and avoid (see Lee et al., 2015 for parameter settings for particular behavioral patterns). The parameter values used in our experimental study are summarized in Table 2, where the agent is set to behave either to exchange rewards or to rush consistently. These values were decided based on optimized parameters in Lee et al., 2015. The learning rate η was set to 5/6 for η*f*(*a*(*k*)) term to be 0.5.

**TABLE 2:** Parameter configuration of the self-concept fairness model for different behavior.

**Behavioral pattern**	**α**	**β**	**γ**	**θ**
Mutual exchange	0.986	0.069	0.110	0.710
Rush	0.863	−0.739	0.673	0.696

The second module handles the user interface (UI) of the game. In the current UI, we designed a simple graphic display for the purpose of behavioral studies. The UI also includes functions to receive a player’s input and apply it to the game. We used a standard computer keyboard to receive inputs in this study, but different kinds of UI devices can be readily connected. Other UI functions are updating and displaying rewards to both players. While rewards are updated every iteration, one can determine how often the players are informed of their rewards. In our experiment, we display rewards at the end of every iteration. The UI allows an experimenter to set up hyperparameters to run the game, including the duration of an iteration, the frequency of changing the position of an object, and the payoff setting. For instance, in our experiment, we set the duration of a single iteration as 3 s, the frequency of changing objects’ position as 1 s and the payoff setting as the one in Table 1.

**FIGURE 1:**
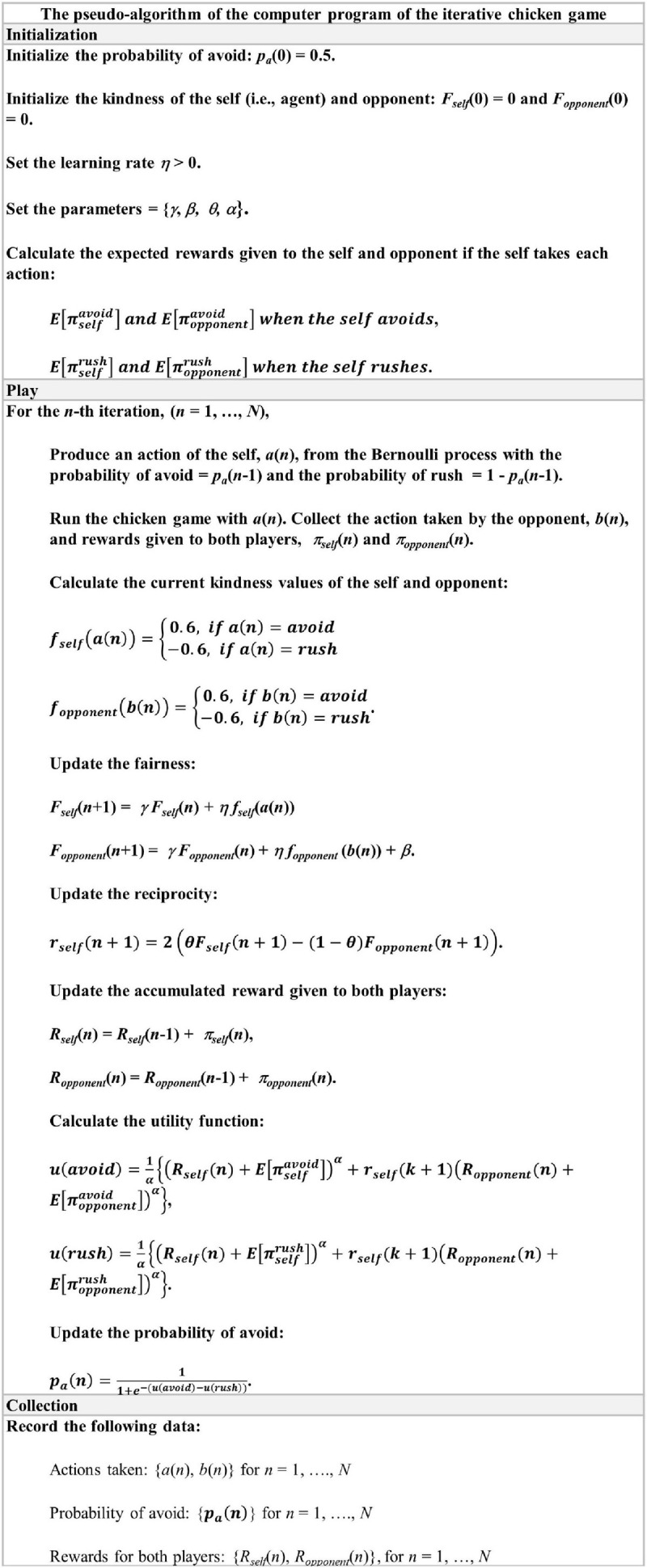
The pseudo-algorithm of the computer program to run the iterative chicken game with an artificial agent making a decision based on the self-concept fairness model (see the text). Here, the self indicates the artificial agent and the opponent can be any player.

The third module includes the algorithm for the decision process of the artificial agent. The module basically consists of three components: Initialization, Play, and Collection. The Initialization component initializes all the required parameters for the self-concept fairness model as well as variables and learning rate. The Play component determines the behavior of the artificial agent at every iteration until the predetermined number of iterations is completed. The Collection component records all the data generated from the game for further analyses. The pseudo-code of this algorithm is illustrated in Figure 1.

### Experiment

We performed an experiment where people played the iterative chicken game against the artificial agent developed in this study, in order to demonstrate that one can use this artificial agent to study human behavior in competitive economic games. To this end, we examined whether changes in the agent’s behavioral patterns could induce changes in human players’ behavior. It would demonstrate a possibility to generate bilateral joint actions between a human player and the artificial agent for a further analysis. We also examined whether human players behaved differently when they perceived an opponent as a human or computer player. Differential behavioral outcomes would demonstrate a difference in the sense of fairness when playing against human and computer players.

#### Participants

Twenty healthy university students participated in this study (ten females). All participants had normal or corrected-to-normal vision with no history of neurological, major medical, or psychiatric disorder. Informed consent was provided by every participant. The study was conducted according to the Declaration of Helsinki, with the approval of the Ulsan National Institute of Science and Technology (UNIST) IRB committee (UNISTIRB-15-04-C). Participants were randomly assigned to one of the two groups: a human-opponent group and a computer-opponent group (see below). Each group thus included ten participants. The data of two participants in the human-opponent group were excluded in the analysis due to error during data collection.

#### Task

**FIGURE 2:**
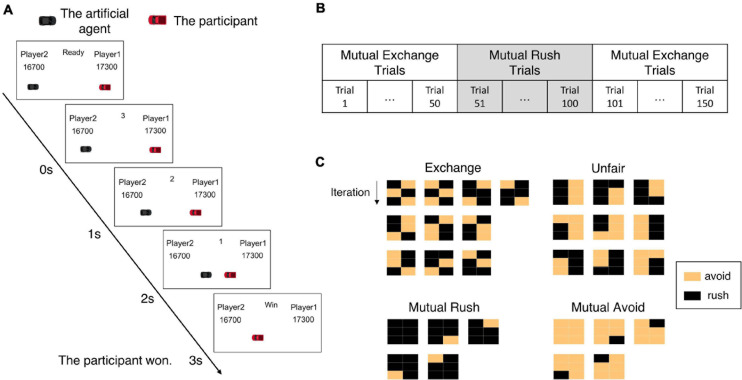
The experimental paradigm. **(A)** The procedure of the iterative chicken game. In each iteration, two cars appear on the screen and approach toward each other for 3 s. One of the cars is controlled by the artificial agent and the other by an experiment participant. The game result and payoffs are displayed at the end of the iteration. The displayed game result indicates whether the participant wins or loses. In this example, the artificial agent avoided but the participant did not, so the participant won. **(B)** The experiment contains a total of 150 iterations among which the artificial agent plays in a direction to mutually exchange rewards with the opponent in the first 50 iterations, then in another direction to keep rushing in the second 50 iterations, and returning to the same direction as the first in the last 50 iterations. **(C)** Bilateral joint action patterns from both the participant and the artificial agent are analyzed in the window of 3 consecutive iterations. Here are shown possible bilateral action patterns belonging to each of the four behavioral patterns: mutual rush, mutual avoid, unfair, and mutual exchange. Each matrix indicates a bilateral action pattern; a column stands for a player and a row for an iteration. The yellow box represents the behavior of avoidance and the black one means rush.

Each participant was informed of the procedure to play the iterative chicken game. Afterward, participants in the human-opponent group were informed that they would play against the experimenter who would play the game in the next room. In contrast, participants in the computer-opponent group were informed that they would play against a computer player. However, both groups actually played against the artificial agent developed in this study. Before the start of the game, participants initially received 20,000 points and were instructed to maximize their income. Participants iteratively played the chicken game 150 times against the artificial agent without a break. In the beginning of each trial (iteration), the game display was presented where two cars appeared at each end of the horizontal lane along with the text showing a ready state. Then, the game started with the disappearance of this “ready” signal. During the game, the two cars approached toward each other for 3 s. The left car was controlled by the artificial agent while the right one controlled by the participant. The cars moved toward each other in a discrete manner, changing their positions to predetermined locations at every second. The time information was also displayed to participants. If both cars rushed until the end of the game, each car would change its position three times from the beginning point to the crashing point (see Figure 2A).

Participants could make a decision to rush or avoid anytime during the game period. Participants were instructed to press the spacebar on the keyboard if they decided to avoid or pressed nothing if they decided to rush. If participants or the artificial agent decided to avoid, it affected the car immediately by making the car invisible and terminating the iteration.

To investigate how a human participant adapts to sudden changes in fairness represented in the opponent’s behavior during the chicken game, we segregated the whole trials into three phases containing 50 trials each (Figure 2B). In the first phase, the artificial agent played with the set of parameters tuned to generate fair behavior, emphasizing mutual exchange of rewards between players. With these parameters, the artificial agent tended to avoid in the current trial if it rushed and earned reward in the previous trial and *vice versa*. In the second phase, the artificial agent immediately changed its parameter values set to generate unfair behavior, with a much higher probability of rush regardless of previous rewards and fairness evaluation. In the third phase, the artificial agent immediately turned back to its parameter setting of the first phase. We intended to observe how participants react to these sudden changes in fairness in the opponent’s behavior.

### Data Analysis

We collected the behavioral data of participants paired by corresponding behavioral outcomes of the artificial agent. Each participant yielded 150 data samples that included action choice (rush = 0 or avoid = 1) and decision-making time (0–3 s). We also collected the time course of points that participants earned over iterations.

To analyze joint behavior between participants and the artificial agent, we defined four behavioral patterns according to the previous study (Lee et al., 2015). The first pattern was a “mutual exchange” pattern where each player took the turn to avoid while the opponent rushed at every trial. The second was a “mutual rush” pattern where both players consistently rushed and the third was a “mutual avoid” pattern where both consistently avoided. The last was an “unfair” pattern where one kept rushing while the other kept avoiding. We investigated joint action outcomes between participants and the artificial agent within a window of three consecutive iterations to determine which pattern the windowed bilateral actions belonged to. More specifically, we categorized the windowed bilateral actions into four different patterns as follows: (1) mutual exchange pattern if two players exchanged avoid/rush over at least two iterations; (2) mutual rush pattern if two players mutually rushed toward each other for at least two iterations; (3) mutual avoid pattern if two players mutually avoided for at least two iterations, and (4) unfair pattern if one kept rushing and the other kept avoiding consecutively over at least two iterations. If the windowed action outcomes did not match any of the four patterns, we regarded it as “undefined.”

Figure 2C illustrates the examples of each behavioral pattern except for “undefined.”

We slid the window by one iteration in each phase and restarted the window in the beginning of a new phase. Then, we counted the number of occurrences of each pattern in each phase for each participant. For each pattern, we set the count of that pattern as a dependent variable (DV), and phase (1st, 2nd, and 3rd) and group (human-opponent and computer-opponent) as independent variables (IVs). Then, we performed a two-way analysis of variance (ANOVA) on the count of each pattern. To find how well participants returned to their original behavioral playing patterns in the third phase, we compared the count of each pattern between the first and the third phase using a paired *t*-test for each group.

To analyze the decision-making time, we measured the timing of pressing the spacebar by participants in each trial (0–3 s) and calculated the mean value in each phase. Since participants pressed the space bar only when they decided to avoid, the decision-making time analyzed here only reflects the decision to avoid. We applied the two-way ANOVA to the mean decision-making time with the same factors as phase and group.

We also analyzed the scores each participant earned over iterations. Since the score was accumulated over iterations, we focused only on the final score after each phase. Again, we applied the two-way ANOVA to the final score with the same factors as phase and group.

## Results

### The Iterative Chicken Game Outcomes

**FIGURE 3:**
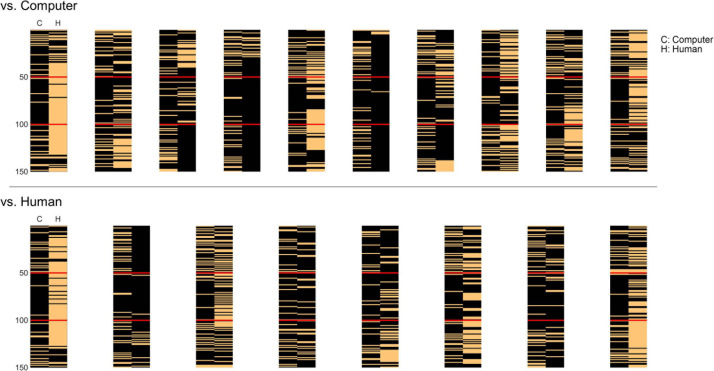
The behavioral patterns over one hundred fifty iterations during the chicken game of all eighteen participants. In the top panel are the patterns of the group knowing they played against a computer opponent and in the bottom panel are those of the group knowing they played against a human opponent. The left column in each pattern represents the artificial agent’s behavior (C) and the right column represents that of the human player (H). The temporal order of iterations starts from the top to the bottom rows in each pattern. The black pixel denotes rush and the orange one denotes avoid actions. The red lines divide the iterations into three phases: the agent played by exchanging turns of rush/avoid (1–50 iterations); by rushing (51–100); and by exchanging again (101–150).

The developed artificial agent played the iterative chicken game program against a total of eighteen human players, generating actions of rush or avoid over one hundred fifty iterations in each player. The agent basically played in the same manner (i.e., the same parameter settings) against all participants, although its actual actions could be different across participants because of the agent’s intrinsic probabilistic action generation process and its dependency upon the previous actions of the self and human opponents. Thus, we observed individual patterns of action outcomes for each participant (Figure 3). Note that the action patterns of the agent drastically changed to rush entering into the second phase (from the 51st iteration), and returned to exchange entering into the third phase (from the 101st iteration). Yet, individual human players showed a variety of behavioral patterns in response to these changes. Nonetheless, we focused our analysis on finding behavioral differences between the two groups of participants during the game.

**FIGURE 4:**
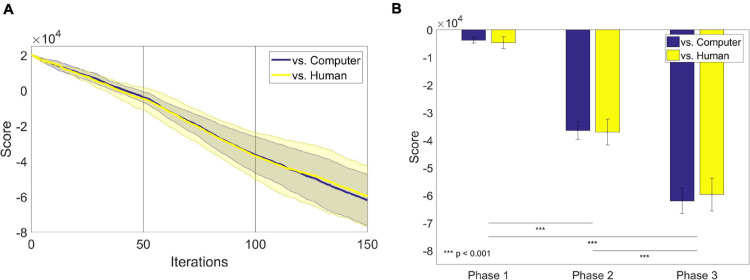
**(A)** The average scores over one hundred fifty iterations in each group: computer-opponent (blue) and human-opponent (yellow). The shadings denote the standard error of mean. The initial score was given as 20,000. **(B)** The average scores earned by participants after the last iteration of each phase.

We also traced the scores that participants received for every iteration (Figure 4A). Generally, the score gradually decreased as the game went on. There was no apparent difference in the trace of scores between the groups. We also compared the slope made by the scores along iterations. We defined the slope of each participant at each phase as the difference between the last and the first score of each phase divided by the number of iterations of each phase, 50. For the slope of the score, a two-way ANOVA showed the main effect of the phase [*F*(2,48) = 10.77, *p* = 0.0001]. There was no main effect of the group [*F*(2,48) = 0.21, *p* = 0.65]. A Tukey-Kramer *post-hoc* analysis revealed that the slopes of the first and the second phase were significantly different (*p* = 0.0007), and the slopes of the second and the third phase are also significantly different (*p* = 0.0005). We further investigated the scores earned by each participant after the last iteration of each phase (Table 3). A two-way ANOVA with the factors of group and phase revealed the main effect of phase on scores only [*F*(2,548) = 107.07, *p* = 2.0189 × 10^–18^; Figure 4B].

**TABLE 3:** The scores earned by each participant (S1 – S18) after the last iteration of each phase.

**vs. Computer**				**vs. Human**			
	**Phase 1**	**Phase 2**	**Phase 3**		**Phase 1**	**Phase 2**	**Phase 3**
S1	500	−17,100	−36,400	S9	−2,400	−17,900	−35,000
S2	−9,200	−51,300	−77,100	S10	−4,000	−39,600	−65,000
S3	5,400	−20,200	−42,700	S11	−6,200	−43,300	−68,600
S4	−9,400	−45,700	−73,700	S12	−8,700	−50,200	−83,300
S5	−12,800	−48,500	−74,300	S13	−6,700	−34,500	−57,100
S6	−800	−32,300	−50,000	S14	−4,500	−48,000	−79,800
S7	−7,100	−47,700	−74,600	S15	−4,900	−40,900	−70,600
S8	−4,000	−33,600	−48,300	S16	−900	−35,900	−56,800
				S17	−1,400	−31,500	−52,200
				S18	1,400	−22,900	−51,100
AVG	−4,675	−37,050	−59,638	AVG	−3,830	−36,470	−61,950

*The average (AVG) was calculated for each phase in each group: computer-opponent (left) and human-opponent (right). The initial score was 20,000.*

### Behavioral Changes During the Iterative Chicken Game

**FIGURE 5:**
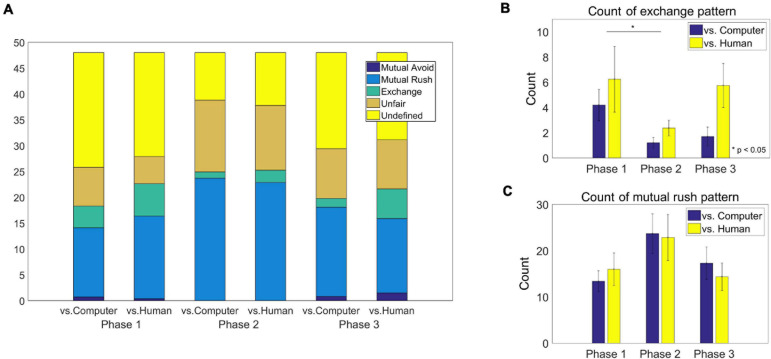
**(A)** The average count of behavioral pattern categories of each group in each phase. The four categories of behavioral patterns includ the mutual avoid, mutual rush, exchange and unfair patterns (see text for details). Behavioral patterns other than these categories were marked as undefined. **(B)** The average frequency of the exchange pattern of each group in each phase. There was a significant difference in count between two groups (*p* = 0.0329) and between the first phase and the second phase (*p* = 0.0374). **(C)** The average frequency of the mutual rush pattern of each group in each phase. There was a main effect of the phase (*p* = 0.0484).

We investigated the count of each of the four bilateral behavioral patterns, including exchange, mutual rush, mutual avoid and unfair pattern, as well as undefined ones (see section “Data analysis”). The results of each category in each phase are listed in Appendix A. The distribution of count in each phase is illustrated in Figure 5A. Since the artificial agent took actions with parameters tuned to the exchange or the mutual rush patterns, we focused on the count of the exchange and the mutual rush patterns in each phase. For the frequency of the exchange pattern, a two-way ANOVA showed the main effects of both group [*F*(1,48) = 4.82, *p* = 0.0329] and phase [*F*(2,48) = 3.25, *p* = 0.0475], respectively, but no interaction effect (Figure 5B). The human-opponent group generated the exchange behavioral pattern more often with the computer agent (4.79 times/phase) than the computer-opponent group did (2.37 times/phase). As expected, the exchange pattern significantly decreased in the second phase (1.79 times/phase), where the artificial agent was most likely to rush all the time, compared to the first phase (5.23 times/phase). The count of the exchange pattern increased again in the third phase (3.73 times/phase). For the count of the mutual rush pattern, a two-way ANOVA showed the main effect of phase [*F*(2,48) = 3.23, *p* = 0.0484] but no group effect. Participants in both groups tended to mutually rush more often against the opponent in the second phase (23.29 times/phase) compared to the first phase (14.7 times/phase) and the third phase (15.84 times/phase; Figure 5C).

## Discussion

In this study, we developed a method to investigate human behavior during a competitive economic game. Specifically, we developed an artificial agent that could play the iterative chicken game with a human player based on the self-concept fairness model. The agent made a decision to avoid or rush in the chicken game by computing the fairness of both an opponent and the self. We built a computer program based on Matlab to implement this artificial agent in the iterative chicken game, along with a set of parameters that controlled the agent’s behavior toward rush, avoid or exchange. Using this program, one can gather and analyze the behavioral data of human players against the agent with a specific parameter setting. To validate that the developed agent could induce changes in human players’ behavioral patterns, we performed an experiment in which we altered the parameter settings of the agent’s behavior in a direction from exchanging to rushing in the middle of the game. The behavioral result showed that human players’ behavioral patterns were changed by this alteration in the agent’s behavior. We also demonstrated a case to utilize the developed method to study human behavior by dividing the participants into two groups and informing each group that they played against a computer or a human. The analysis of human behavioral data revealed that the group aware that they are playing against humans exhibited more exchanging patterns than the other group that was aware of playing against the computer, indicating that players became more sensitive to fairness when competing against humans. These results suggest that the developed artificial agent in the iterative chicken game may be useful to study human behavior in competitive social interactions.

A number of studies have investigated human behavior during participation in the chicken game where participants played the game against a computer opponent. Some studies utilized a computer-based player but did not employ a computational model that dynamically chooses an action in response to human players’ actions (Fukui et al., 2006; Wang et al., 2013, 2017). Others developed computational models to make a decision for an artificial agent by simulating neural circuits of decision-making (Zaldivar et al., 2010; Asher et al., 2012). In contrast, our method generates an artificial agent that plays the game based on computational models for human behavior. The agent’s action selection is derived from the self-concept fairness model that can elucidate human behavior in the iterative chicken game. Moreover, our method offers the means to adjust the artificial agent’s playing strategy by tuning several parameters. In doing so, one can drive the agent to play more aggressively or cooperatively and observe how human opponents respond.

It is also plausible to create an agent with artificial intelligence and let it learn how to play the chicken game based on reinforcement learning, similar to those developed to play a variety of interactive games (van den Dries and Wiering, 2012; Silver et al., 2016, 2018). Yet, there are several advantages to building an artificial agent based on analytical models of human behavior without learning from data. First, it can provide a parametric model for the agent such that one can systematically control the agent’s behavior by adjusting the model parameters. Second, the analytical model can provide a framework for the development of agents with various desired properties. Third, it is straightforward to analyze the agent’s behavior precisely based on the model’s structure and functions. Fourth, theoretical backgrounds of the analytical models would make it possible to investigate social constructs such as emotions, fairness, and distance by incorporating them into the models. Nonetheless, it will be worth integrating the problem-solving power of artificial neural networks with the analytical models to implement rich repertoires of decision making processes in the artificial agent of the chicken game (Liu et al., 2018).

In our human experiment, we demonstrated using the developed method to study human behavior in social interactions. In particular, we observed different behavioral patterns in human participants when they recognized an opponent as a human or as a computer. Using economic games other than the chicken game, previous studies have also investigated human behavior and corresponding neural mechanisms when humans played the game with robots or other humans. Using the classical prisoner’s dilemma game, Hegel et al. (2008) demonstrated that human participants reported experience of more fun with, feeling better in the face of winning of, and attribution of more intelligence to an opponent when the opponent exhibited more anthropomorphic features. This research team also showed that the medial prefrontal cortex and right temporoparietal junctions, which belong to the core network of theory of mind (Schurz et al., 2014), were more activated in interaction with more human-like opponents (Krach et al., 2008). Rosenthal-von der Putten et al. (2019) investigated neural activity when humans evaluated artificial agents and made a decision about them; they found that temporoparietal junctions and dorsomedial prefrontal cortical activity represented human-likeness of the artificial agent and ventromedial prefrontal cortical activity represented the subjective likability of the artificial agent. Unlike the previous studies on the human-likeness of artificial agents, our study developed an artificial agent that could continuously interact with a human player through the chicken game by updating its concept of fairness, providing a means to study behavioral and neural mechanisms in the sense of fairness in social interaction with artificial partners.

We observed that players tended to generate exchange behavioral patterns more when they perceived an opponent as human than when they perceived an opponent as a computer. This was supported by the significant difference between groups in the count of the exchange pattern and the observation that the number of counts of the exchange pattern among four behavioral patterns was twice more in the human-opponent group than in the computer-opponent group in our experiment (see section “Behavioral changes during the iterative chicken game”). Although we could not observe the difference in overall game performance between groups, differences in behavioral patterns related to fairness may provide insights to human behavior and support the advantage of using the proposed artificial agent in the iterative chicken game.

In our experiment, we defined four behavioral patterns: exchange, mutual rush, mutual avoid, and unfair pattern. The patterns such as exchange pattern need two trials to be defined, which can be multi-trial patterns and patterns such as mutual rush need only one trial to be defined, which can be stated as single-trial pattern. However, we used the way of counting which was to slide windows of three iterations with a step size of one iteration. This might raise an issue of under-counting of multi-trial patterns relative to single-trial patterns. In order to verify that this does not happen, we have conducted an additional analysis by simulating the counting of behavioral patterns using randomly generated behavioral data. We repeated 10,000 times of generating 50 behavior data of both players on 50% probability of avoid or rush and counting the number of behavioral patterns that appeared. The average count of four behavioral patterns is 3.7(±2.5) for mutual avoid, 3.8(±2.6) for mutual rush, 7.5(±3.4) for exchange and 6.7(±3.1) for unfair pattern. The count of the exchange and unfair pattern was almost twice larger than that of mutual avoid and mutual rush. This result shows that the multi-trial patterns (e.g., mutual exchange) are not under-counted, rather counted more, and this might be due to more number of occasions of possible patterns. On the other hand, in the results, exchange patterns appeared far less than mutual rush patterns, so this result seems meaningful.

The method proposed in this study can be used in various ways to study human behavioral patterns in social interactions. Although the present study developed an artificial agent that makes a decision depending on fairness, the developed algorithm can be modified to drive the agent’s decision based on other social and cognitive attributes such as emotion (Cox et al., 2007) or social values (Wang et al., 2017). In addition, our method will be useful to investigate how various factors influence the perception of fairness in competitive interactions such as opponent gender (Vermeulen et al., 2014), in- and out-of-network opponents (Fareri and Delgado, 2014), efficiency (Ben-Asher et al., 2013), payoff structure (Cabon-Dhersin and Etchart-Vincent, 2012), and social identity (Wit and Wilke, 1992). Since our algorithm runs iteratively with adjustable parameters, which enables the modeling of dynamics of the artificial agent’s behavior over iterations, it can be used to take a close look at the emergence of specific social behavior, including reciprocity (Browning and Colman, 2004), temporal discounting (Stephens et al., 2002), and retaliation (Asher et al., 2012).

The present study developed an artificial agent based on computational models of human behavior in the iterative chicken game and demonstrated its utility to study human players’ behavioral patterns in different situations. Yet, the current method is in the beginning stage of the development of such agents; more diverse and multi-faceted psychological characteristics may be added to the current version. In addition, the current model bases a decision merely on self-concept fairness, but other attributes to decision-making in competitive social interactions should be also considered to depict social behavior more precisely. The four parameters included in the current model would also be limited in illustrating the variability of human behavioral patterns. This could be one of the reasons why there was a considerable portion of “undefined” patterns in our experimental data (see Figure 5). A more comprehensive model for behavioral patterns should be developed in the future to explain more sophisticated joint behavioral patterns manifested in the iterative chicken game. Furthermore, even though we observed that the counts of a specific behavioral pattern, which was the exchange pattern in the results, was significantly different between the human-opponent and computer-opponent group, the difference of players’ behavioral patterns between conditions – playing against human and against computer opponents – was not seemingly large. Because the goal of this study was to verify the feasibility of using the newly developed artificial agent to investigate dynamic behavioral patterns of human players, we believe that the results still can support this feasibility. However, more in-depth investigations on human behavior with a larger number of participants should be conducted in the follow-up studies.

## Data Availability Statement

The raw data supporting the conclusions of this article will be made available by the authors, without undue reservation. The MATLAB code for the program used in the experiment is available at https://github.com/MJKIM28/chickengame.

## Ethics Statement

The studies involving human participants were reviewed and approved by Ulsan National Institute of Science and Technology. The patients/participants provided their written informed consent to participate in this study.

## Author Contributions

S-PK designed the study, analyzed the data, and wrote the manuscript. MK conducted the experiment, analyzed the data, and wrote the manuscript. JL conducted the experiment and analyzed the data. YC and O-SK oversaw the study. All authors read and approved the final manuscript.

## Conflict of Interest

The authors declare that the research was conducted in the absence of any commercial or financial relationships that could be construed as a potential conflict of interest.

## Figures and Tables

**FIGURE B1:**
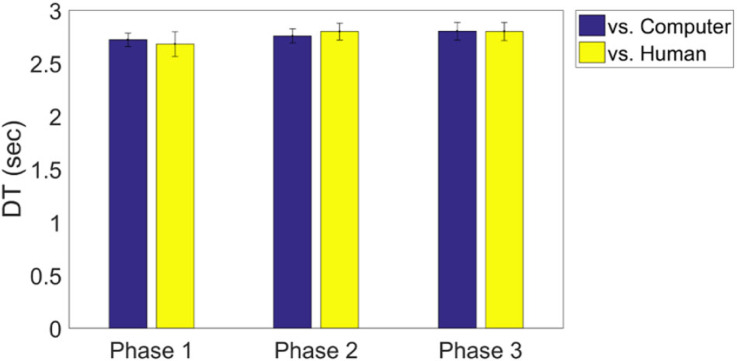
The average decision-making time (DT) of each group (human-opponent and computer-opponent) in each phase. Note that participants should respond within 3 s if they decide to avoid.

## Footnotes

1 https://github.com/MJKIM28/chickengame

## Appendix

### Appendix A

**TABLE A1:** The average number of times each of the four behavioral pattern categories (mutual avoid, mutual rush, and exchange and unfair) appeared in each of the three phases (1, 2, and 3).

		**Mutual avoid**	**Mutual rush**	**Exchange**	**Unfair**	**Undefined**
Phase 1	vs. Computer	0.7	13.4	4.2	7.5	22.2
	vs. Human	0.375	16	6.25	5.25	20.125
Phase 2	vs. Computer	0	23.7	1.2	13.9	9.2
	vs. Human	0	22.875	2.375	12.5	10.25
Phase 3	vs. Computer	0.8	17.3	1.7	9.6	18.6
	vs. Human	1.5	14.375	5.75	9.5	16.875

*Note that the sum of each row is equal to 48 as we identified a behavioral pattern category by sliding a 3-iteration window over 50 iterations with a sliding step of 1.*

### Appendix B

We examined the decision-making time for avoiding in each phase. The mean decision-making time in each group is summarized in Table B1. A two-way ANOVA revealed no significant difference between groups or between phases (*ps* > 0.05; Figure B1).

**TABLE B1:** The average decision-making time (DT) for avoiding of each group in each phase.

		**DT (s)**
Phase 1	vs. Computer	2.7225
	vs. Human	2.6821
Phase 2	vs. Computer	2.7577
	vs. Human	2.8002
Phase 3	vs. Computer	2.8041
	vs. Human	2.8016
